# *Piper sarmentosum* Effects on 11β-Hydroxysteroid Dehydrogenase Type 1 Enzyme in Serum and Bone in Rat Model of Glucocorticoid-Induced Osteoporosis

**DOI:** 10.3390/molecules21111523

**Published:** 2016-11-15

**Authors:** Siti Fadziyah Mohamad Asri, Elvy Suhana Mohd Ramli, Ima Nirwana Soelaiman, Muhamad Alfakry Mat Noh, Abdul Hamid Abdul Rashid, Farihah Suhaimi

**Affiliations:** 1 Department of Anatomy, Faculty of Medicines, Universiti Kebangsaan Malaysia Medical Centre (UKMMC), Jalan Yaacob Latif, Bandar Tun Razak, 56000 Cheras, Kuala Lumpur, Malaysia; elvysuhana@yahoo.com.my; 2 Department of Human Anatomy, Faculty of Medicine and Health Sciences, Universiti Putra Malaysia, 43400 UPM Serdang, Selangor, Malaysia; 3 Department of Pharmacology, Faculty of Medicines, Universiti Kebangsaan Malaysia Medical Centre (UKMMC), Jalan Yaacob Latif, Bandar Tun Razak, 56000 Cheras, Kuala Lumpur, Malaysia; imasoel@ppukm.ukm.edu.my; 4 Department of Anatomy, Faculty of Medicine, Universiti Malaya Medical Centre (UMMC), 59100 Lembah Pantai, Kuala Lumpur, Malaysia; pae2300@yahoo.com; 5 Anatomy Unit, Basic Medical Science Cluster, Faculty of Medicine, Universiti Teknologi MARA (UiTM), Sungai Buloh Campus, Jalan Hospital, 47000 Sungai Buloh, Selangor, Malaysia; dahar_219@yahoo.com

**Keywords:** glucocorticoid, osteoporosis, Piper sarmentosum, 11β-hydroxysteroid dehydrogenase

## Abstract

Glucocorticoid-induced osteoporosis is one of the common causes of secondary osteoporosis. *Piper sarmentosum* (*Ps*) extract possesses antioxidant and anti-inflammatory activities. In this study, we determined the correlation between the effects of *Ps* leaf water extract with the regulation of 11β-hydroxysteroid dehydrogenase (HSD) type 1 enzyme activity in serum and bone of glucocorticoid-induced osteoporotic rats. Twenty-four Sprague-Dawley rats were grouped into following: G1: sham-operated group administered with intramuscular vehicle olive oil and vehicle normal saline orally; G2: adrenalectomized (adrx) control group given intramuscular dexamethasone (120 μg/kg/day) and vehicle normal saline orally; G3: adrx group given intramuscular dexamethasone (120 μg/kg/day) and water extract of *Piper sarmentosum* (125 mg/kg/day) orally. After two months, the femur and serum were taken for ELISA analysis. Results showed that *Ps* leaf water extract significantly reduced the femur corticosterone concentration (*p* < 0.05). This suggests that *Ps* leaf water extract was able to prevent bone loss due to long-term glucocorticoid therapy by acting locally on the bone cells by increasing the dehydrogenase action of 11β-HSD type 1. Thus, *Ps* may have the potential to be used as an alternative medicine against osteoporosis and osteoporotic fracture in patients on long-term glucocorticoid treatment.

## 1. Introduction

Long-term glucocorticoid therapy induces osteoporosis and has become the most common cause of secondary osteoporosis. Bone loss induced by pharmacological glucocorticoids can occur even with a very low dose and progresses at a fast rate, depending on the dose and duration of treatment [1]. Trabecular bone is more severely affected than cortical bone, increasing the risk of fracture [2,3].

11β-hydroxysteroid dehydrogenase (11β-HSD) isoenzymes play an important role in the regulation of corticosteroid hormonal action. Due to their pre-receptor action [4], the two isoenzymes of 11β-HSD catalyze the interconversion of hormonally-active cortisol in humans (corticosterone in rodents) to inactive cortisone in humans (11-dehydrocorticosterone in rodents) [5]. 11β-HSD type 1 is a low-affinity nicotinamide adenine dinucleotide phosphate (NADPH)-dependent bidirectional enzyme that can interconvert inactive cortisone to active cortisol, predominantly as a reductase enzyme. In contrast, 11β-HSD type 2 is a high-affinity nicotinamide adenine dinucleotide (NAD)-dependent unidirectional enzyme with dehydrogenase action and inactivates cortisol to cortisone. Reductase activity for physiological glucocorticoids has not been seen for 11β-HSD type 2. Reductase activity of 11β-HSD type 1 enzyme is responsible for the local generation of glucocorticoids in bone, and is expressed in human osteoblasts and osteoclasts. Eijken et al. [6] reported that there is an inverse relationship between 11β-HSD type 1 enzyme activity and osteoblast differentiation after continuous treatment of human osteoblast cells with dexamethasone.

The blood concentration ratio of cortisone/cortisol might be an index to assess the systemic activity of 11β-HSD enzyme [7]. The result shows a status of cortisol–cortisone shuttle in serum and gives an evidence for the dehydrogenase activity of 11β-HSD type 2 enzyme that irreversibly converts the active cortisol into inactive cortisone. However, 11β-HSD type 1 is the predominant isozyme, with 11β-HSD type 2 being virtually undetectable in cultures of primary human osteoblasts and cells from normal adult bone (osteoclasts and osteoblasts) [8]. 11β-HSD type 2 enzyme is highly expressed in mineralocorticoid target tissues like adrenal, kidney, colon, and placenta [9]. For bone study, cloning of rat 11β-HSD type 2 protein has been used, since it has an exclusive dehydrogenase activity [10,11]. A rat model of glucocorticoid-induced osteoporosis (GIO) has been used by many researchers to assess the action of endogenous and exogenous glucocorticoids in osteoblastic and osteoclastic differentiation pathways [12,13].

*Piper sarmentosum*—also known as “daun kaduk”—is a creeping terrestrial herb, usually used for flavoring local cuisine in South East Asia. The methanolic extract of *Piper sarmentosum* possesses naringenin, a very potent natural antioxidant that shows high superoxide scavenging activity [14]. Many studies have shown that *Piper sarmentosum* possesses pharmacological properties, such as anti-tuberculosis [15], anti-cancer [16], hypoglycaemic [17], antimalarial [18], antioxidant [14], neuromuscular blocking [19], anti-atherosclerotic [20], and anti-inflammatory activities [21].

Our previous studies on *Piper sarmentosum* water extract at 125 mg/kg/day [22,23] showed the pharmacological properties of promoting fracture healing in the osteoporotic state. Ramli et al. [24] did a study of the effect of *Piper sarmentosum* water extract of the same dose on bone markers and its relationship with the stress enzyme 11β-hydroxysteroid dehydrogenase type 1, and found out that there was a reduction in the expression of 11β-HSD type 1 enzyme and an increment in the dehydrogenase activity of the enzyme in the femoral bone of adrenalectomized rats on long-term dexamethasone treatment.

This study aimed to assess the activity of 11β-HSD type 1 enzyme by measuring the serum level of 11-dehydrocorticosterone and corticosterone, the femur corticosterone level, and the protein expression of 11β-HSD type 1 in the femoral bone of glucocorticoid-induced osteoporotic rats. The body weight of the Sprague-Dawley rats was also monitored throughout the study. We were aiming to see changes of the activity of 11β-HSD type 1 after two months of treatment with *Piper sarmentosum* water extract.

## 2. Results

### 2.1. Changes in the Serum Level of Corticosterone

**Figure 1:**
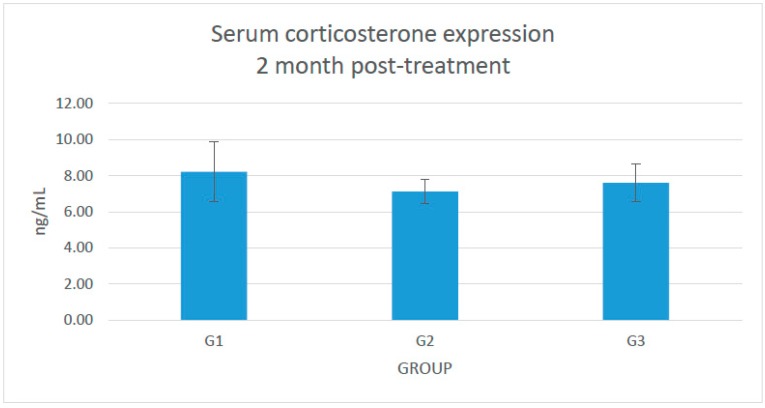
The effect of serum corticosterone expression in G1: sham-operated control; G2: adrenalectomized (adrx) control given intramuscular dexamethasone (DEX) 120 μg/kg/day; and G3: adrx and given intramuscular DEX 120 μg/kg/day and *Piper sarmentosum* 125 mg/kg/day.

No significant changes in the serum corticosterone expression were seen (Figure 1).

### 2.2. Changes of Corticosterone Level in the Femoral Bone

**Figure 2:**
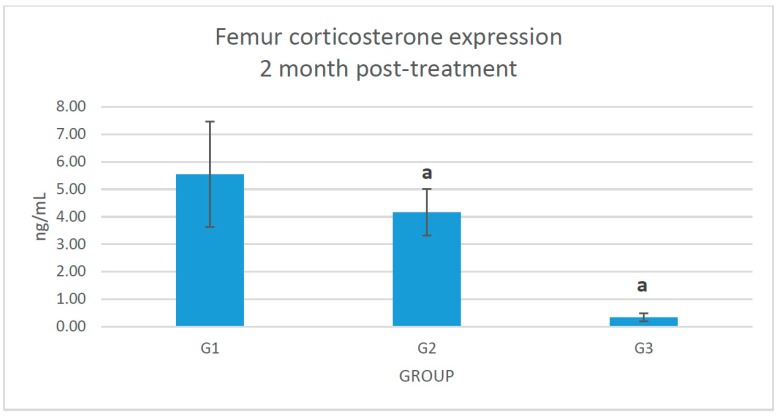
The effect of femoral corticosterone expression in G1: sham-operated control; G2: adrenalectomized (adrx) control given intramuscular dexamethasone (DEX) 120 μg/kg/day; and G3: adrx and given intramuscular DEX 120 μg/kg/day and *Piper sarmentosum* 125 mg/kg/day. Identical letter (a) indicates significant difference between groups at *p* < 0.05.

There was a significant reduction of corticosterone expression in the femur bone of glucocorticoid-induced osteoporotic rats treated with *Piper sarmentosum* (G3) compared to adrenalectomized control group (G2) (Figure 2).

### 2.3. Changes of 11β-HSD Type 1 Protein Expression in the Femoral Bone

**Figure 3:**
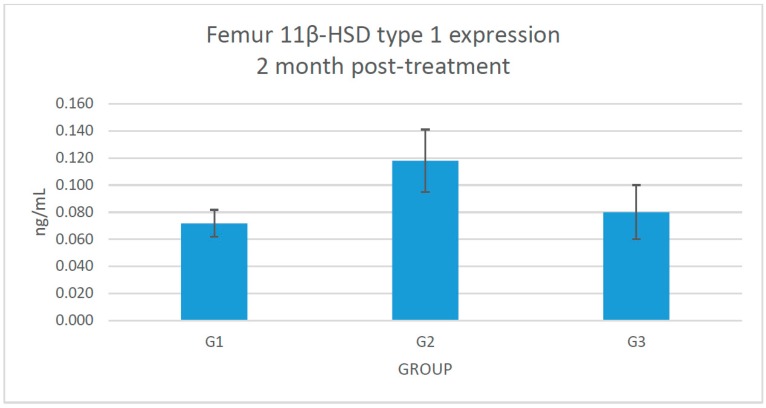
Graph shows the effect of femoral 11β-hydroxysteroid dehydrogenase (HSD) type 1 protein expression in G1: sham-operated control; G2: adrenalectomized (adrx) control given intramuscular dexamethasone (DEX) 120 μg/kg/day; and G3: adrx and given intramuscular DEX 120 μg/kg/day and *Piper sarmentosum* 125 mg/kg/day.

No significant changes were seen in the 11β-HSD type 1 protein expression in the femoral bone (Figure 3).

### 2.4. Changes of Rats’ Body Weight

**Figure 4:**
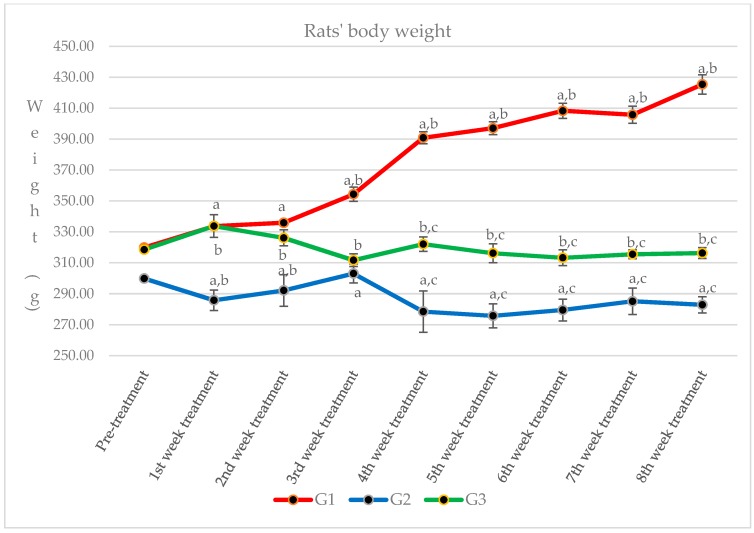
The changes of rats’ body weight in G1: sham-operated control; G2: adrenalectomized (adrx) control given intramuscular dexamethasone (DEX) 120 μg/kg/day; and G3: adrx and given intramuscular DEX 120 μg/kg/day and *Piper sarmentosum* 125 mg/kg/day. Identical letters (a,b,c) indicate significant difference between groups at *p* < 0.05.

We observed the rats’ body weight throughout the study (Figure 4), and we found significant changes between sham-operated rats (G1) compared to both glucocorticoid-induced osteoporotic rats treated with *Piper sarmentosum* (G3) and the adrenalectomized control group (G2). There was also a significant decrement of body weight in Group 2 compared to Group 3.

## 3. Discussion

In this study, the rats were adrenalectomized to remove the endogenous glucocorticoids which are influenced by the circadian rhythm and stress [25]. The endogenous glucocorticoids were replaced by dexamethasone to ensure a constant level of glucocorticoid in the rat. Normal saline ad libitum was given to maintain the normal sodium homeostasis. Long-term dexamethasone treatment reduced the bone mineral density, calcium content, and femur length of the adrenalectomized rats [26].

This study showed that *Piper sarmentosum* had a correlation with the 11β-HSD type 1 local activity in the femur bone. There is no systemic correlation with the 11β-HSD type 1 activity in the serum. The serum level of corticosterone and 11-dehydrocorticosterone were undetectable before starting treatment (data not provided) because of the adrenalectomy. After 2 months, the serum level of corticosterone was detected, probably because of the effect of reductase activity of 11β-HSD type 1 at the cellular level, which converts the inactive 11-dehydrocorticosterone to corticosterone. However, we did not measure the femoral 11-dehydrocorticosterone concentration. There is also a possibility of extra adrenal secretion of corticosterone, which is still under debate [27]. The adrenalectomized control rats exposed to 120 μg/kg/day dexamethasone revealed a significant increment (*p* < 0.05) of femoral corticosterone after 2 months. Adrenalectomized rats that were given *Piper sarmentosum* leaf extract had significantly reduced (*p* < 0.05) femoral corticosterone concentration, and significantly increased body weight (*p* < 0.05). 11β-HSD type 1 protein expression in the femoral bones was not significantly increased in the adrenalectomized control group, in comparison with the adrenalectomized rats given *Piper sarmentosum* leaf extract. This might be due to the short course of treatment or maybe insufficient dose of the *Piper sarmentosum*. The rats’ body weight showed that adrenalectomized rats on dexamethasone had very low weight. The sham rats revealed significant increment of body weight, in comparison with the adrenalectomized control rats and adrenalectomized rats given *Piper sarmentosum* leaf extract. Therefore, it shows that there were changes of the bone density where the *Piper sarmentosum* prevented further loss. Our previous study showed that increasing 11β-HSD type 1 dehydrogenase activity and reduction in 11β-HSD type 1 expression in the bone leads to inhibition of bone resorption in glucocorticoid-induced osteoporotic rats given *Piper sarmentosum* leaf extract [24]. Based on the biomechanical strength test and structural histomorphometric analysis, our earlier study also revealed that *Piper sarmentosum* had improved bone strength with better trabecular thickness, volume, and number [28]. In another study, we found that there was a discrete 1.1-fold reduction of the 11β-HSD type 1 mRNA expression in the adrenalectomized dexamethasone-treated rats given *Piper sarmentosum* [29]. This finding was consistent with our earlier studies that postulated that *Piper sarmentosum* water extract is a potential 11β-HSD type 1 inhibitor via switching of the 11β-HSD type 1 activity from reductase to dehydrogenase [30]. The current results showed that *Piper sarmentosum* had the potential to protect the bone against glucocorticoid-induced osteoporosis by acting locally at the bone cells, thus lowering the harmful effect of active corticosterone.

The active glucocorticoid (cortisol in humans and corticosterone in rodents) exerts an important physiological role. The genomic effects of glucocorticoids are mediated via the glucocorticoid receptor (GR). 11β-HSD type 1 has bidirectional action that interconverts inactive cortisone in humans (11-dehydrocorticosterone in rodents) and active cortisol in humans (corticosterone in rodents). It is believed to function as a reductase-generating active glucocorticoid at a pre-receptor level, enhancing glucocorticoid receptor activation. 11β-HSD type 1 generates active cortisol from its inactive form (cortisone), such that intracellular levels of active glucocorticoid are dependent not only on circulating concentrations, but also upon tissue-specific activation. Dehydrogenase activity has been reported in some intact cell systems, with the enzyme directionality varying according to the differentiation status of the particular cell type [31]. The effects of glucocorticoids on bone occur through multiple pathways, including reduced intestinal calcium absorption, renal calcium loss, reduced sex steroid levels, and reduction in muscle [32]; however, the most important effects are on bone formation and resorption which are coordinated by osteoblasts forming bone and osteoclasts breakdown bone [33]. High circulating levels of glucocorticoids impair bone formation by directly impacting osteoblast function, reducing proliferation and differentiation, and increasing the rates of apoptosis [34]. 11β-HSD type 1 expression is primarily noted to be localized to osteoblasts, but some expression is also seen in osteoclasts in human adult bone [8]. It is possible that the effects on osteoclastic bone resorption were mediated indirectly through the inhibition of 11β-HSD1 in osteoblasts [35].

The extracts of different parts of *Piper sarmentosum* are known to have potential benefits. *Piper sarmentosum* leaf extract is rich with naringenin, a natural antioxidant compound with superoxide scavenging activity [14]. Both aqueous and boiled aqueous extraction of *Piper sarmentosum* revealed high phenolic content [36]. *Piper sarmentosum* exerts beneficial effects on osteoporotic fracture healing, as assessed by quantitative and qualitative radiological analyses [23]. Histologically, *Piper sarmentosum* gives beneficial effects on endochondral ossification in osteoporotic fracture in ovariectomised rats [37]. In another study, *Piper sarmentosum* improved the biomechanical strength of bone, and also the structural histomorphometry changes in glucocorticoid-induced osteoporotic rats [28]. Ramli et al. [12] reviewed potential beneficial effects of *Piper sarmentosum* as a new hope for the treatment of osteoporosis. The use of aqueous extraction of *Piper sarmentosum* up to 2000 mg/kg/day did not show subacute toxicity in the Sprague-Dawley rats [38].

Therefore, we may conclude that *Piper sarmentosum* had a positive role in controlling the changes in glucocorticoid-induced osteoporotic bone. Thus, *Piper sarmentosum* supplement is a potential alternative medicine that can be used in patients on long term glucocorticoid therapy. However, the mechanisms involved need further exploration to thoroughly clarify the pharmacological pathway.

## 4. Materials and Methods

### 4.1. Preparation of Piper sarmentosum Water Extract

*Piper sarmentosum* plants (Voucher No. UKMB 29851) were identified by the Herbarium Unit, Faculty of Science and Technology, National University of Malaysia. The leaves were taken and dried in an oven with constant airflow. Then, the leaves were ground and boiled with water. The aqueous solution was sent to the Herbal Technology Centre, Forest Research Institute of Malaysia (FRIM) for freeze drying to obtain the powdered form. *Piper sarmentosum* powder (125 mg/kg) was dissolved in normal saline and administered orally.

### 4.2. Animals and Treatment

All procedures were carried out according to the ethical clearance from the UKM Research and Animal Ethics Committee (PP/ANAT/2010/ELVY/14-JULY/313-JULY-2010-MAY-2012). Twenty-four Sprague-Dawley rats weighing 250–300 g were obtained from the Animal Breeding Centre, National University of Malaysia [39]. They were divided into three groups of eight rats and given the following treatment: G1, sham-operated control group administered with intramuscular vehicle olive oil and vehicle normal saline via oral gavage; G2, adrenalectomized (adrx) control group given intramuscular dexamethasone (120 μg/kg/day) [28] and vehicle normal saline via oral gavage; and G3, adrx group given intramuscular dexamethasone (120 μg/kg/day) and water extract of *Piper sarmentosum* (125 mg/kg/day) [24] via oral gavage. Adrenalectomy was done two weeks after receiving the animals. The animals were first anaesthetized with Ketapex and Xylazil. Dorsal midline and bilateral flank muscle incisions were made, and the adrenal glands were identified and removed. The incisions were sutured, and Poviderm cream was applied daily to prevent infection and aid wound healing. The rats were also given intramuscular injection of the antibiotic Baytril 5%, for 5 days post-adrenalectomy. The sham-operated rats were also incised at the dorsal midline and bilateral flank muscle. The adrenal glands were identified and left in situ before the incisions been sutured. The treatment was started two weeks post-adrenalectomy. Dexamethasone [26,40] was dissolved in olive oil and administered intramuscularly at 120 μg/kg/day, six days a week, for eight weeks. *Piper sarmentosum* (125 mg/kg/day) was dissolved in normal saline and administered orally once daily, six days a week, for eight weeks. The animals were kept in clean cages under natural sunlight during daytime and darkness at night. They had free access to rat pellets. The sham-operated group was given tap water ad libitum, while adrenalectomized rats were given normal saline ad libitum [26]. The rats’ body weight were taken every two days.

### 4.3. Sample Collection

The rats were put under mild diethyl ether anaesthesia for serum collection the day before starting the treatment, at one month, and at two months after supplementation with *Piper sarmentosum*. Between 9 a.m. and 11 a.m., the rats were placed in a transparent glass desiccator, under a screen to avoid direct contact with cotton-soaked with diethyl ether. The rats were monitored inside the desiccator with a tightly closed lid. When there was no response to toe pinch, the rats were removed from the desiccator for the retro-orbital blood sampling [41]. At sacrifice, the right femoral bones were cleared of surrounding tissues and wrapped with gauze that had been dipped in PBS at pH 7.4. The bones and serum were frozen at −80 °C until analysis.

### 4.4. Measurement of Parameters

The metaphyseal area of the femoral heads were homogenized with metal beads in 2 mL microtubes. The phosphate buffered saline (PBS) with a pH of 7.4 was used as a homogenizing solution. The samples were centrifuged at 5000 rpm for 5 min at 4 °C. The supernatants were analysed using the sandwich-type ELISA kit for rats (*Rattus norvegicus*) from USCN Life Science Inc. (Wuhan, China) for detection of 11β-HSD type 1 protein expression. The samples were added to microtiter plates which were pre-coated with an antibody specific to the 11β-HSD type 1. A biotin-conjugated antibody preparation specific for 11β-HSD type 1 was added. Next, avidin conjugated to horseradish peroxidase (HRP) was added and incubated. After adding tetramethylbenzidine (TMB) substrate solution, only samples that contained 11β-HSD type 1, biotin-conjugated antibody, and enzyme-conjugated avidin exhibited colour change. These enzyme–substrate reactions were terminated by sulphuric acid solution, and the colour changes were measured spectrophotometrically at 450 nm wavelength. The serum was prepared for analysis of corticosterone and 11-dehydrocorticosterone concentration level [42] by using ELISA kits from MyBioSource, Inc. (California, CA, USA). Blood serum (2 mL) was taken through the retro-orbital sinus. After 20 min at room temperature, the serum was centrifuged at 3000 rpm for 20 min. The supernatant was taken for measurement of the corticosterone and 11-dehydrocorticosterone concentration levels using indirect method of ELISA. The intra-assay and inter-assay coefficient of variations (CV) for 11β-HSD type 1 and corticosterone ELISA kits were <10% and <12%, respectively. The intra-assay and inter-assay CVs for 11-dehydrocorticosterone were <15%. No significant cross-reactivity or interference were observed between corticosterone, 11-dehydrocorticosterone, and 11β-hydroxysteroid dehydrogenase type 1, with their analogues.

### 4.5. Statistical Analysis

Data were tested for normality using the Shapiro–Wilk test. The data was analysed by parametric statistics (i.e., the ANOVA test), followed by post hoc Tukey’s test for comparison between various treatment groups using the Statistical Package for Social Sciences (SPSS) software version 22 (IBM Software, New York, NY, USA). Data was presented as mean ± standard error of the mean, and *p* < 0.05 was considered statistically significant.
